# Nitric oxide in plants: an assessment of the current state of knowledge

**DOI:** 10.1093/aobpla/pls052

**Published:** 2012-12-20

**Authors:** Luis A. J. Mur, Julien Mandon, Stefan Persijn, Simona M. Cristescu, Igor E. Moshkov, Galina V. Novikova, Michael A. Hall, Frans J. M. Harren, Kim H. Hebelstrup, Kapuganti J. Gupta

**Affiliations:** 1 Institute of Environmental and Rural Science, Aberystwyth University, Edward Llwyd Building, Aberystwyth SY23 3DA, UK; 2 Life Science Trace Gas Facility, Molecular and Laser Physics, Institute for Molecules and Materials, Radboud University, PO Box 9010, 6500 GL Nijmegen, The Netherlands; 3 Timiryazev Institute of Plant Physiology, Russian Academy of Sciences, ul. Botanicheskaya 35, Moscow 127276, Russia; 4 Department of Molecular Biology and Genetics, Section of Crop Genetics and Biotechnology, Aarhus University, Forsøgsvej 1, DK-4200 Slagelse, Denmark; 5 Department of Plant Sciences, University of Oxford, South Parks Road, Oxford OX1 3RB, UK

## Abstract

**Background and aims:**

After a series of seminal works during the last decade of the 20th century, nitric oxide (NO) is now firmly placed in the pantheon of plant signals. Nitric oxide acts in plant–microbe interactions, responses to abiotic stress, stomatal regulation and a range of developmental processes. By considering the recent advances in plant NO biology, this review will highlight certain key aspects that require further attention.

**Scope and conclusions:**

The following questions will be considered. While cytosolic nitrate reductase is an important source of NO, the contributions of other mechanisms, including a poorly defined arginine oxidizing activity, need to be characterized at the molecular level. Other oxidative pathways utilizing polyamine and hydroxylamine also need further attention. Nitric oxide action is dependent on its concentration and spatial generation patterns. However, no single technology currently available is able to provide accurate *in planta* measurements of spatio-temporal patterns of NO production. It is also the case that pharmaceutical NO donors are used in studies, sometimes with little consideration of the kinetics of NO production. We here include *in planta* assessments of NO production from diethylamine nitric oxide, *S*-nitrosoglutathione and sodium nitroprusside following infiltration of tobacco leaves, which could aid workers in their experiments. Further, based on current data it is difficult to define a bespoke plant NO signalling pathway, but rather NO appears to act as a modifier of other signalling pathways. Thus, early reports that NO signalling involves cGMP—as in animal systems—require revisiting. Finally, as plants are exposed to NO from a number of external sources, investigations into the control of NO scavenging by such as non-symbiotic haemoglobins and other sinks for NO should feature more highly. By crystallizing these questions the authors encourage their resolution through the concerted efforts of the plant NO community.

## Abstract (precis)

Nitric oxide (NO) is a plant signal contributing to plant stress responses and development. We here review some of the key advances in this field but also highlight certain key aspects of plant NO biology that require further attention.

## Introduction

Nitric oxide (NO) emerged as a signal in plants during the last decade of the 20th century and has since come to be associated with a large number of phenomena. These have been extensively reviewed in a recent series of special reviews (Wendehenne and Hancock 2011). However, although impressive progress has been made, some persistent questions remain unanswered. Most fundamentally, various means of NO generation have been described, but the contexts of when and where they are deployed need a fuller description. In particular, the underlying basis of frequently reported arginine-oxidizing ‘nitric oxide synthase’-like activity requires final resolution. It must also be admitted that NO detection methods are often inadequate and that the accuracy and tissue specificity of methods used for measuring *in planta* NO production can be questioned. We also suggest that more attention should be paid to understanding how NO metabolism in plants interferes with and contributes to the larger scale nitrogen cycle. The purposes of this review are to highlight the progress made in plant NO research to date and to point out areas where further work is required.

## Nitric oxide: some major themes from animal research

Nitric oxide has long been a major research topic in animals and some important aspects of this work merit a brief outline, thereby allowing a comparison with the current understanding in plants. The NO story in mammals started when it was noted that treatment of cultured macrophages with bacterial lipopolysaccharides (LPS) resulted in the production of NO (Hauschildt *et al.* 1990). Nitric oxide has emerged as an important component of innate resistance mechanisms elicited by pathogens or endotoxins such as LPS. In the innate resistance, NO generated by phagocytes may be directly antimicrobial via DNA damage or disruption of iron–sulfur (4Fe–4S) enzymes (Nathan 1995). Another important driver for NO research is its role in the regulation of smooth muscle contraction as its signalling pathways are pharmaceutical targets to mitigate the effects of cardiac infarctions (Murad 2004). This important role for NO grew out of observations that the vasodilatory effects of neurotransmitters such as acetylcholine were only observed when the endothelium cells covering the smooth muscle of the vessel wall were retained. Screens for soluble endothelium-derived relaxing factors led to the identification of NO as well as prostacyclin and endothelium-derived hyperpolarizing factor. To promote vasodilation, NO diffuses into smooth muscle cells to bind to iron centres within the active site of a soluble guanylate cyclase complex which generates cGMP from GTP. The soluble forms of guanylate cyclase are complexes of α and β subunits, each unit ranging between 79 and 80 kDa, and each with a haem site and catalytic domains. cGMP activates protein kinase G, which in smooth muscle suppresses calcium influxes to reduce calcium-dependent muscle contraction. The effects of this NO-initiated pathway can be reversed through the activity of phosphodiesterases, which convert cGMP to GMP (Derbyshire and Marletta 2012). These phosphodiesterases are targets for Viagra (sildenafil) and their inhibition leads to a more persistent NO effect (Moreland *et al.* 1999).

In animals, an important mechanism of NO generation is the deamination of arginine by nitric oxide synthase (NOS) to form citrulline and NO. Nitric oxide synthase is homologous to P450 cytochrome *c* reductases, its activity being dependent on the reductant NADPH, flavin mononucleotide, flavin adenine dinucleotide (FAD) and tetrahydrobiopterin. The NOS group of enzymes is usually sub-classified as Ca^2+^/calmodulin-activated brain NOS (nNOS), endothelial NOS (eNOS) and inducible NOS (iNOS). These NOS are associated, respectively, with the neuronal, smooth muscle relaxation and induction following immunological challenge (Forstermann and Sessa 2012). Besides NOS, mammalian tissues can generate NO through the reduction of NO_2_^−^ in the mitochondrion via reduction at complex III or cytochrome *c* oxidase (complex IV) (Shiva 2010) or enzymes with nitrate reductase (NR) activity that may be xanthine oxidoreductases (XOR) (Jansson *et al.* 2008).

## Plants join the NO party

Nitric oxide first came to prominence within the context of regulating plant defence during plant–pathogen interactions (Delledonne *et al.* 1998; Durner *et al.* 1998). Nitric oxide has been implicated in defence against *Pseudomonas syringae* pathogens (Delledonne *et al.* 1998; Clarke *et al.* 2000; Mur *et al.* 2005), in barley infected with powdery mildew and downy mildew on pearl millet (Prats *et al.* 2005; Manjunatha *et al.* 2009) or *Botrytis cinerea*-challenged *Arabidopsis* (Mur *et al.* 2012). As with mammalian systems, bacterial LPS, a contributor to pathogen-associated molecular patterns triggered immunity (PTI), proved to be a highly effective initiator of NO (Zeidler *et al.* 2004). Given these plant responses, it is unsurprising that many pathogens have evolved genes that could suppress NO-associated event(s). For example, *Erwinia chrysanthemi* expresses the flavohaemoglobin (fHb) *HmpX*, which oxidizes NO to NO_3_^−^ (Boccara *et al.* 2005). In other cases, the pathogen may actively elicit host NO to aid in the infection process. For example, the virulence factor cryptogein produced by the oomycete *Phytophthora cryptogea* aids pathogenesis by promoting host cell death via NO generation (Foissner *et al.* 2000; Lamotte *et al.* 2004). In addition, pathogen-generated NO can promote the formation of key fungal infection structures (Prats *et al.* 2008; Turrion-Gomez and Benito 2011). Thus, depending on the pathogenic lifestyle, NO can act as either a pathogen virulence or a host defence factor (Perchepied *et al.* 2010; Turrion-Gomez and Benito 2011; Mur *et al.* 2012; Rasul *et al.* 2012).

Nitric oxide also plays an important role in symbiotic organisms, particularly between legumes and *Sinorhizobium* (Baudouin *et al.* 2006). Nitric oxide was first detected complexed to leghaemoglobin (within nitrogen-fixing nodules of cowpea and pea; Kanayama and Yamamoto 1991). Transcriptional analyses of legumes suggested that the NO played an early role in nodule development, being observed as early as during infection thread development within root hairs, through which *Sinorhizobium* colonizes the host (del Giudice *et al.* 2011). Indeed, NO may induce the expression of flavanoids which are essential in establishing bacterial *nod* gene expression, which initiates root hair deformations and represents one of the earliest stages of the symbiotic interaction (Dénarié *et al.* 1992). Categorical proof of an important role for NO in *Sinorhizobium* interactions with its host was obtained from *Medicago truncatula* plants expressing an NO-oxidizing fHb gene regulated by a nodule-specific promoter. Following inoculation with *Sinorhizobium meliloti* strain, nodule formation was significantly delayed. Transcriptional analysis indicated that the host gene *MtCRE1*, which encodes a cytokinin receptor, and the cell cycle-switching gene *MtCCS52A* were suppressed in expressing nodules (del Giudice *et al.* 2011). *MtCCS52A* triggers selected cells within the root primordium to switch from mitotic cycles to endoreduplicating cycles (where genomes duplicate without cell division), which is essential for nodular symbiotic cell differentiation in *M. truncatula* (Vinardell *et al.* 2003). Moving beyond nitrogen fixation, NO generation also plays a role in symbiotic interactions involving arbuscular mycorrhizal fungi (Calcagno *et al.* 2012).

It is immediately obvious, even in this brief overview, that NO seems to be involved in a series of apparently incongruous events; it is involved in host defence (both cell death and PTI), pathogen virulence and also many forms of symbiotic interaction. This poses a question that is not only relevant to plant pathology: How exactly could NO fulfil these very different roles? It seems probable that the relative concentration of NO is important (Beligni and Lamattina 1999; Turrion-Gomez and Benito 2011) but equally, cell-, tissue- or organelle-specific roles are vital, reflecting interactions with differing components and signalling pathways.

Spatio-temporal subtlety, as well as key roles for interactions with hormone signalling, is also a feature of the developmental actions of NO. Excellent examples of the role of NO in root development have been described in a series of papers produced by the Lamattina group. Thus, NO is required for root organogenesis (Pagnussat *et al.* 2002), the formation of adventitious roots (Pagnussat *et al.* 2003), lateral root development (Correa-Aragunde *et al.* 2004) and root hair formation (Lombardo *et al.* 2006). The role of auxin is very well established in various features of root development (Kramer and Bennett 2006) so that an important advance was made when NO and cGMP were implicated as downstream effectors of at least some auxin effects (Pagnussat *et al.* 2003). In adventitious root formation, auxin (indole acetic acid, IAA) was suggested to act through NO to activate mitogen-activated protein kinase (MAPK) signalling (Pagnussat *et al.* 2004) and through a modification of the auxin receptor TIR1 (Terrile *et al.* 2012). Ultimately, NO appears to influence root development through the initiation of cell cycle genes and patterns of cellulose synthesis (Correa-Aragunde *et al.* 2006, 2008), and influencing vesicle trafficking in root hair formation (Lombardo and Lamattina 2012). At the root apices, NO has been shown to influence the arrangement of the actin cytoskeleton (Yemets *et al.* 2011).

At the cross-roads between developmental and abiotic stress tolerance lies the regulation of the stomatal aperture by NO (Hancock *et al.* 2011). Early work showed that NO was produced in stomata and was an output of well-characterized abscisic acid (ABA) signalling pathways (Neill *et al.* 2002). Thus, an ABA-induced increase in cytoplasmic pH acts together with H_2_O_2_ to initiate NO generation. In *Vicia faba* guard cells, NO regulates Ca^2+^ release from intercellular Ca^2+^ stores, which regulates inward-rectifying K^+^ channels to close stomata (Garcia-Mata *et al.* 2003; Bright *et al.* 2006; Wilson *et al.* 2009). However, NO can appear to be a redundant element in stomatal regulation as in, for instance, conditions of rapid dehydration (Ribeiro *et al.* 2009). It is apparent that H_2_O_2_ effects can stimulate and at least partially parallel the effects of NO in *Arabidopsis* (Bright *et al.* 2006). This redox-sensitive step appears to involve the ethylene receptor ETR1, adding yet another level of NO hormone interactions at the stomatal level (Desikan *et al.* 2006). Additionally, NO concentration appears to be important in its effects in plants, with high amounts opening stomata (Sakihama *et al.* 2003). These effects were linked to NO effects on outward-rectifying K^+^ channels which are Ca^2+^ insensitive, possibly by direct modification of the K^+^ channel by NO (Sokolovski and Blatt 2004).

A finer level of spatial effects is demanded when considering the differential intracellular role of NO. Various cellular compartments such as mitochondria (Gupta *et al.* 2009), peroxisomes (Corpas *et al.* 2009) and chloroplasts (Jasid *et al.* 2006) have been shown to produce NO. It is very probable that NO has a specific role in each compartment, possibly interacting with local signal events. For example, NO has recently been shown to modulate mitochondrial alternative oxidase activity to influence the generation of reactive oxygen species (ROS), net NO production and shift primary metabolism towards amino acid biosynthesis via inhibition of aconitase (Cvetkovska and Vanlerberghe 2012; Gupta *et al.* 2012). Another role for NO was indicated by the work of Palmieri *et al.* (2008), who showed that NO produced during plant–pathogen interactions can inhibit the P protein of glycine decarboxylase (GDC) activity by *S*-nitrosylation (see below) to promote the hypersensitive response (HR). In this case GDC inhibition could limit NADH to the electron transport chain and leads to a change in redox of the electron transport chain of mitochondria. In the peroxisome, Ortega-Galisteo *et al.* (2012) recently showed that the NO that is produced in peroxisomes nitrosylates proteins such as catalase and glyoxylate oxidase, which are involved in photorespiration, β-oxidation and the detoxification of ROS.

Taking some lessons from these papers reveals the need for a careful experimental design that needs to be considered by plant NO scientists. In particular, as far as possible, strategies should be followed where the subtlety of NO effects is not lost. An example of this could be through the use of pharmaceutical NO donors, which represent an easy method to apply NO exogenously. If the NO concentration hypothesis is correct, these should be coupled to a better means of visualizing and/or measuring *in situ* NO generation (see below). Experimentally, it is preferable to utilize approaches where the spatial, temporal and biochemical features of NO action in the biological phenomena under investigation are preserved. Admittedly, this is easier for such as stomata and root hairs but should be a major driver in experimental design for all NO biologists.

## How NO is generated in plants?

As NO is now firmly established as an important signal in plant science, a remaining task is to describe the various mechanisms of NO generation in plants. After some wrong turns this is now much less of a contentious issue. Guo *et al.* (2003) identified a novel NOS in *Arabidopsis* which had the same co-factor requirements as NOS but exhibited no significant sequence homology to the mammalian form. The derived mutant *atnos1* has proven to be a useful tool in NO research as it does display reduced NO production, and has been used to show the roles of NO in floral development (He *et al.* 2004) or the interaction of NO with ROS (Zhao *et al.* 2007). However, subsequently AtNOS1 was found not to possess NOS activity and was renamed AtNOA1 (Nitric Oxide Associated1; Zemojtel *et al.* 2006) and indeed has been established to be a GTPase (Moreau *et al.* 2008).

Currently, a seemingly bewildering number of sources for NO (at least seven) have been identified (Gupta *et al.* 2011*a*). Any discomfort that arises from this is most likely due to a comparison with mammalian cells, where NOS represents a bespoke NO-generating system with different forms having well-defined roles and expression patterns. In the apparent absence of a true plant NOS, it may be better to consider the varieties of ROS generation as a better paradigm for understanding NO production. Thus, although ROS generation is most often linked to NADPH oxidases, other sources, peroxidases, polyamine oxidases and non-enzymatically from photosynthetic and respiratory electron transport chains, have important roles (Wojtaszek 1997).

A series of reductive pathways for NO generation have been described (Gupta *et al.* 2011*a*), including a peroxisomally located XOR which reduces nitrite to NO at the expense of NADH under anaerobic conditions (Corpas *et al.* 2008) or a plasma membrane-bound nitrite: NO reductase (Ni-NOR) (Stöhr *et al.* 2001). However, it is cytosolic NR that is rapidly emerging as the main source of NO in plants under aerobic conditions. Nitrate reductase is implicated in the NO production during bacterially induced defence (Modolo *et al.* 2005), disease development in certain pathogenic interactions (Shi and Li 2008), drought (Freschi *et al.* 2010), cold (Zhao *et al.* 2009), stomatal regulation (Srivastava *et al.* 2009) and many developmental processes, for example, the initiation of flowering (Seligman *et al.* 2008). Nitrate reductase is a cytosolic enzyme that undergoes a regulatory switch from its preferential high-affinity substrate NO_3_^−^ (*K*_m   __nitrate_ = <40 μM) to NO_2_^−^ (low affinity; *K*_m__nitrite_ = 100 μM) and producing NO. An important question to consider is how this switch comes about (Gupta *et al.* 2011*a*). Currently, regulation seems to be at the level of substrate competition so that high nitrite levels are required to competitively inhibit NO_3_^−^ reduction. This could come about through either increased NO_3_^−^ influx into the vacuole or efflux from the cell. In *Arabidopsis*, accumulation into the vacuole involves AtCLCa (*Arabidopsis thaliana* Chloride Channel a) which acts as a proton antiporter (Geelen *et al.* 2000). Thus, it is possible that NO_3_^−^ vacuolar import is promoted by cytoplasmic pH changes, possibly driven by tonoplastic H^+^-ATPases (De Angeli *et al.* 2007). This could enable the build-up of a 50-fold excess of nitrate in the vacuole compared with the cytoplasm (De Angeli *et al.* 2007). An alternative/additional mechanism could be to promote NO_3_^−^ efflux from the cells. In the case of the HR elicited by cryptogein in tobacco cells, nitrate efflux was shown to be vital to cell death and defence. This resulted in a rapid 60 % drop in the concentration of internal NO_3_^−^ (Wendehenne *et al.* 2002). Concomitant with NO_3_^−^ efflux is an influx of calcium, on which NO production was completely dependent (Lamotte *et al.* 2004) and acts via calmodulin/calmodulin-like proteins (Ma *et al.* 2008). The longer-term generation of calcium is influenced by NO (Lamotte *et al.* 2006) and this integrates Ca^2+^–NO effects into the web of positive and negative feedback loops that typify plant–pathogen interactions (Robert-Seilaniantz *et al.* 2011). In terms of triggering events, it seems likely that NO_3_^−^ efflux is one of a series of calcium ion-mediated events which contribute to plasma membrane depolarization and also causing the extrusion of K^+^ ions and water loss, all of which are early features of HR-type cell death (Garcia-Brugger *et al.* 2006). Clearly, more work is needed on the regulation of NO_3_^−^ fluxes in disease responses and more widely, and especially the possible perturbation that could occur when plants are supplied with high levels of nitrogen fertilizer must be assessed.

As well as modulation of nitrogen flux within cells, direct regulation of NR itself should be considered. Nitrate reductase is a relatively labile protein, the levels of which are based on its relative expression and degradation. Thus, NR may be phosphorylated at a serine residue to interact with 14–3–3 proteins to inactivate the protein and, mostly likely, promote proteolysis (Kaiser and Huber 2001). However, the phosphorylation of NR is Ca^2+^ dependent for at least three types of kinase, and the formation of the inactive form is influenced by divalent cations (Weiner and Kaiser 1999, 2000). Thus, we need to understand why Ca^2+^ influxes which activate NO_3_^−^ efflux and NO production do not also encourage NR degradation. This could be via the action of Type 2A phosphatases (Deruere *et al.* 1999) or, interestingly, hexose sugars via unknown mechanisms (Cotelle *et al.* 2000).

One other reductive mechanism of NO generation merits mention as it allows NO production under very small partial pressures of oxygen (Planchet *et al.* 2005). This involves a mitochondrial-based NR activity where NO_2_^−^ acts as a terminal electron acceptor for cytochrome *c* oxidase/reductase (Castello *et al.* 2006). This can maintain some ATP generation under hypoxic conditions and will also reduce NO_2_^−^ to generate NO which can be scavenged by non-symbiotic Hbs (Stoimenova *et al.* 2007). The nitrite reductase activity of cytochrome oxidase/reductase is well established under hypoxia (Gupta *et al.* 2011*a*) but may be increasingly important as partial pressures of oxygen are reduced from ambient. The importance of this generational route has been demonstrated by our recent work where we studied NO production under various degrees of hypoxia and identified a threshold at 0.5 % O_2_, below which substantial NO is produced (Hebelstrup *et al.* 2012). This led to the production of ethylene which promotes petiole hypernasty and/or vertical elongation, an important plant mechanism for avoiding the negative effects of submergence in *Rumex palustris* and a wide variety of other species (Voesenek *et al.* 2006).

Moving on to consider oxidative mechanisms of NO generation in plants, there are frequent reports of NOS-like activity in plants, notwithstanding the absence of an isolated enzyme or gene (Corpas *et al.* 2009). Over the last decade the authors have described the NOS activities in peroxisomes (Barroso *et al.* 1999) and chloroplasts (Jasid *et al.* 2006), and in isolated root mitochondria (Gupta and Kaiser 2010). Further, this NOS-like activity shares several of the co-factor requirements for mammalian NOS (Corpas *et al.* 2009). There are also many examples of the use of arginine-based analogues, which inhibit mammalian NOS, to suppress plant NO production, including from our work (Mur *et al.* 2005, 2008). Independent confirmation of this arginine pathway has come from *Arabidopsis* arginase mutants which exhibited increased levels of NO production (Flores *et al.* 2008). Taking all of this work together, we are left with strong data suggesting that an arginine-utilizing pathway can generate NO, although this need not be one that leads to the co-production of citrulline, an important characteristic for true NOS (Tischner *et al.* 2007). The vagueness of this situation begs the question of how specific really is this arginine oxidizing capacity. For example, another oxidative pathway leading to NO generation based on polyamines has been described (Tun *et al.* 2006). The mechanism through which it acts is also unknown, but although very different, mammalian NOS could be a target for mammalian NOS inhibitors (Gupta *et al.* 2011*a*). Given the chequered history of NOS studies in plants, the sources of NOS-like activity in plants and why arginine-based inhibitors are effective in plants need resolving.

Recently, it was discovered that NR-free plant cells are able to oxidize externally supplied hydroxylamine (HA) to NO (Rumer *et al.* 2009*a*, *b*), a pathway that is well characterized in bacteria and animal systems (Vetrovsky *et al.* 1996). Conditions that increase ROS are able to increase the NO production from HA, for instance increasing ROS by mitochondrial complex III inhibitor myxothiazol enhanced NO production from HA (Rumer *et al.* 2009*b*). Interestingly, it was recently shown that isolated non-rice non-symbiotic haemoglobin 1 enzyme could reduce NO_2_^−^ to NO with a rate constant that was far in excess of that reported for Hbs (Sturms *et al.* 2011). Given such an observation, the endogenous production of HA by plants needs to be characterized and the possible enzymatic basis of NO generation from this substrate should also be a target for further research.

## How much NO is made and where?

If the sources of NO generation are now coming to be well characterized, the next problem that needs addressing is the patterns of NO generation. We have already highlighted how several authors are suggesting that the concentration of NO is important for its activity (for example, Beligni and Lamattina 1999; Garcia-Mata *et al.* 2003); therefore, actual measures of *in planta* NO should be a major feature of NO studies. However, as outlined in our recent review (Mur *et al.* 2011), nearly all the approaches currently employed have associated problems.

The most superficially attractive approach is to use diaminofluoresceins (DAF)—fluorescent dyes that are available from many manufacturers. Diaminofluorescein dyes react with N_2_O_3_, a by-product of NO oxidation, with a resulting dramatic increase in fluorescence to allow an approximate quantification of NO content (Kojima *et al.* 1998*a*, *b*). Diaminofluorescein dyes are sensitive NO sensors with detection limits in the nanomolar range and specific to NO as no increased fluorescence was observed with NO_2_^−^, NO_3_^−^, H_2_O_2_ and peroxynitrite (ONOO^−^) (Kojima *et al.* 1998*b*). When used in conjunction with confocal microscopy, DAF dyes offer the possibility of exactly defining the site of NO generation. As suggested above, such a precise tissue resolution is essential in order to begin to assign roles for NO in plant development and in plant–microbe interactions. However, the specificity of DAF dyes has recently been questioned and it is also possible that a differential take-up of the dye by some tissue types or organelles could give artefactual results (Mur *et al.* 2011). For example, Rumer *et al.* (2012) have shown that reactions involving horseradish peroxidase and H_2_O_2_ were sufficient to generate DAF fluorescence *in vitro*. This stated, there is currently no well-established alternative approach to reveal high tissue-specific patterns of NO generation. We therefore expect DAF dyes to be continued to be used. We have therefore recommended that users employing DAF dyes should follow the following steps (Mur *et al.* 2011). First, the background fluorescence of tissues in the absence of the DAF dye should be assessed. Then, most importantly, a fluorescence in the presence of DAF (not the absence of) dyes should be suppressed by co-application of NO scavengers (for example, 2-(4-carboxyphenyl)-4,4,5,5-tetramethylimidazoline-l-oxyl-3-oxide (cPTIO); NO + cPTIO→NO_2_^−^ + cPTI) or inhibitors of mammalian NOS, although this last approach may be questioned. Additionally, we note with interest that newer fluorescent probes have been developed, although these have yet to be widely used by the plant NO community. These include rhodamine B (2-amino-3′,6′-bis(diethylamino)-2,3-dihydrospiro [iso-indole-1,9′-xanthene]-3-one)-based dyes which can act to detect NO_2_^−^ and ONOO^−^ (Rieth and Sasamoto 1998; Yang *et al.* 2002). In a comprehensive assessment of the specificity of rhodamine B dyes, the fluorescence response was greatest with NO and ONOO^−^. However, fluorescence was also significant with hydroxyl radicals (Wu *et al.* 2011), which are likely to be generated in stressed plants (Apel and Hirt 2004). Europium(III) chelate has recently been advanced as an NO fluorescent probe but its wider specificities are yet to be fully assessed (Chen *et al.* 2011).

To be confident in the NO measurements obtained, it is preferable that alternative approaches are employed in parallel. The Griess assay is an indirect colorimetric assay for NO that is available as a kit. In this assay, NO is oxidized to NO_2_*^−^* which reacts with sulfanilic acid and α-naphthylamine under acidic conditions to yield an azo dye. The sensitivity of the Griess assay can be improved by employing a flow-through system where NO emitted from a plant or cell culture is passed via a flow into a receiving vessel where the Griess assay is carried out; the dye accumulates as more NO enters the vessels (Vitecek *et al.* 2008). Another highly popular commercially available means of NO detection from the gas phase is chemiluminescence. The detection system is based on the reactivity of NO with O_3_, which produces excited-state nitrogen dioxide (NO_2_^*^), which emits a photon upon relaxation to the ground state. Therefore each photon is related to a single NO molecule; the NO concentration is then determined by measuring the light intensity. The chemiluminescent approach has been utilized by the present authors and has proved to be a highly sensitive and accurate measure of NO emission from plants (Planchet *et al.* 2005; Mur *et al.* 2011). Its main drawback concerns its lack of selectivity, as molecules such as water can dampen the chemical reaction and lead to erroneous NO measurements. Two additional platforms that we have extensively used to measure NO in the gas phase are laser photoacoustic detection (LPAD) and tunable diode laser absorption spectroscopy (TDLAS) (Mur *et al.* 2005, 2011, 2012), neither of which is currently available in a commercial form. Both target the strongest absorption band of NO centred at 5.3 μm (1876 cm^−1^) (Rothman *et al.* 2005), and use different laser sources such as CO gas lasers, quantum cascade lasers (QCL) or interband cascade lasers. Compared with other methods, laser-based systems directly measure NO molecules and are extremely selective. In LPAD the absorption of NO in bursts of laser light results in pressure variations that generate sound, which is detected by a microphone located within a photoacoustic cell. Traditional LPAD systems require high-power sources and are not user friendly, while compact QCLs are still unable to reach the sub-ppb detection limit (Kosterev *et al.* 2002; Spagnolo *et al.* 2010). However, TDLAS systems are offering the better potential for miniaturization and commercialization. In combination with a thermoelectrically cooled QCL, the key part of TDLAS systems is a multipass absorption cell where the light undergoes multiple reflections between two mirrors. This represents an interaction path length with the NO gas sample of 76 m in a compact design. It allows the detection of NO at and below 1 ppb by volume with a second measuring time (Cristescu *et al.* 2012). Each of these gaseous detection platforms offers the ability to obtain multiple real-time measures of NO production from plants, but there are some important deficiencies. The relationship between the concentration of *in planta* NO and that lost by the plant to the atmosphere is difficult to establish; also it is difficult to measure tissue-specific patterns of NO generation and it is impossible to measure organellar production.

The requirement for accurate measurements of NO production is also important due to the widespread use of chemical NO donors as surrogates for biologically generated NO production in experiments. Many NO donors have been developed, often for pharmaceutical use (Napoli and Ignarro 2003), and are readily obtained commercially. Nitric oxide donors include NONOates (spermidine- or diethylamine-NONOate) (Keefer *et al.* 1996), *S*-nitroso-*N*-acetylpenicillamine (SNAP), *S*-nitrosoglutathione (GSNO) and sodium nitroprusside (SNP). In plant biology NONOate has been used in, for example, the analysis of pathogen-associated cell death (Lamotte *et al.* 2004) and mitochondrial function (Fu *et al.* 2010). Example studies using SNAP to show NO effects have focused on wound healing (Orozco-Cardenas and Ryan 2002), thermotolerance (Xuan *et al.* 2010) and root meristem development by influencing the key auxin effector PIN1 (Fernandez-Marcos *et al.* 2011).

Sodium nitroprusside is frequently used as an NO donor, including by ourselves to validate the LPAD approach (Mur *et al.* 2005) or to investigate the bolting time in *Arabidopsis* (Hebelstrup and Jensen 2008). Others have used SNP to show a role in photomorphogenesis by regulating phytochrome and gibberellin signalling (Lozano-Juste and Leon 2011). Sodium nitroprusside is in fact an NO^+^ donor, which is a highly stable electrophile but can be stabilized by co-ordination with metals (Roncaroli *et al.* 2007). NO^+^ readily forms nitrosothiolate adducts which can be reduced (for example, GSNO + H^+^→GSH + NO^+^) to generate NO gas (Wang *et al.* 2002). An additional problem with SNP is the activity of the ‘spent’ donor (Bethke *et al.* 2006). When assessing the ability of NO to break *Arabidopsis* seed dormancy, it was found that SNP and the ‘spent donor’ products potassium ferricyanide (Fe (III) CN) and potassium ferrocyanide (Fe (II) CN) were all acting via the generation of cyanide (CN^−^). To counter this problem, experimenters must include controls involving the NO ‘spent’ controls (i.e. the remaining products when all NO has been produced).

*S*-nitrosoglutathione is often used as a ‘clean’ NO donor in nitrosylation studies (see below) as it presents no known problem with spent products. This undergoes spontaneous homolytic cleavage of the Cys-based S–NO bond to release NO (Ederli *et al.* 2009). The *S-*nitrosylation process involves initial reaction with O_2_ to form substances such as N_2_O_3_, which dissociates to form the nitrosonium ion, NO^+^. The electrophile NO^+^ will attack thiolate to form S–NO adducts.

**Fig. 1:**
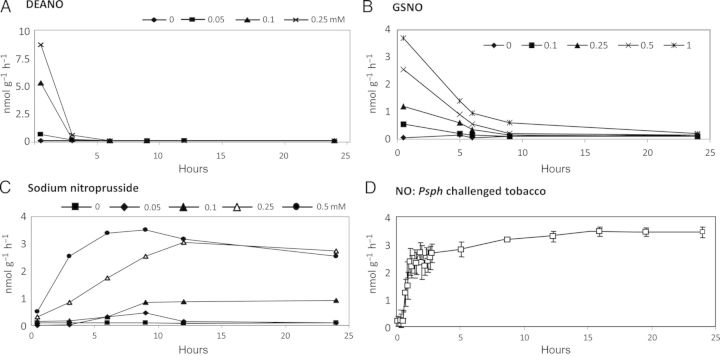
**Nitric oxide production from tobacco leaves infiltrated with NO donor chemicals or *P. syringae* pv. *phaseolicola.*** Nitric oxide production was measured using a QCL from tobacco leaves removed from 5-week-old plants and infiltrated with various concentrations of (A) diethylamine nitric oxide (DEANO); (B) *S*-nitrosoglutathione (GSNO) or (C) sodium nitroprusside and compared with (D) infiltrations with suspensions of 10^6^ cells mL^−1^ 10 mM MgCl_2_ of the HR-eliciting *P. syringae* pv. *phaseolicola* (*Psph*). Results are given as mean NO production: nmol g^−1^ h^−1^ (*n* = 3)±SE. Plant cultivation conditions and bacterial culture are described in Mur *et al.* (2005). Quantum cascade laser measurement protocols are described in Mur *et al.* (2012).

Nitric oxide concentration is clearly important for action but there have been very few attempts to assess the *in planta* kinetics of NO generation by NO donors. Ederli *et al.* (2009) investigated the *in planta* production of NO from SNP, GSNO and injections of mammalian NOS using the oxyhaemoglobin NO assay method. Similarly, we have examined the kinetics of NO production from donors following infiltration of tobacco leaves using our QCL-based approach (Fig. 1). The NO donors diethylamine nitric oxide (DEANO) (Fig. 1A) and SNAP (data not shown) proved to give rise to a short burst of NO. *S*-nitrosoglutathione increased NO for a longer period (Fig. 1B) but SNP gave rise to a more persistent pattern of NO generation (Fig. 1C). This pattern of NO production was similar to that observed during the HR in tobacco elicited by the bacterial pathogen *P. syringae* pv. *phaseolicola*. Taking the above points together, we experimenters need to be circumspect when using NO donors and give thought to *in planta* patterns and amounts of NO production as well as the possible effects of spent donor products.

## How far is there a discrete NO signalling module?

As the mechanisms of NO generation come to be established, the mechanisms of NO signalling are increasingly being resolved. Initial work focused on transcriptomic assessments of NO effects, which revealed the importance of NO-regulated antioxidant responses (Krause and Durner 2004) or the possible roles of iron-deficiency genes in NO-mediated responses to Cd^2+^ (Besson *et al.* 2007). Currently, many groups concentrate on two protein modifications which are specific to NO signalling: thiol protein *S-*nitrosylation and tyrosine nitration, to reveal target signalling components and modes of action. Excellent reviews are available describing the current status of our understanding of the roles of *S-*nitrosylation and nitration (Astier *et al.* 2011; Malik *et al.* 2011; Vandelle and Delledonne 2011) and so we will confine ourselves to a general overview and some general points that emerge.

The regulation of proteins by *S*-nitrosylation is attracting a lot of interest as an important reversible post-transcriptional modification. The following represents some elegant regulatory mechanisms which have recently been reported in the literature. *Nonexpressor of pathogenesis related proteins 1* (NPR1) is a key regulator within the signalling cascade of the key defence hormone salicylic acid. In its non-activated state, NPR1 exists as a cytoplasmically located oligomer with each monomer linked by redox-active disulfide bridges. When activated, cytoplasmic changes lead to reduction of the thiol groups to release monomeric NPR1 forms which are translocated to the nucleus (Mou *et al.* 2003). Apparently, gratuitous translocation of NPR1 into the nucleus leads to proteasome-mediated digestion (Spoel *et al.* 2009). In the nucleus, NPR1 interacts with a range of TGA-class transcription factors, some of which are involved in binding to *as-1* elements to activate *pathogenesis-related protein 1* gene expression. Thus, the oligomeric status of NPR1 is essential to its action and *S-*nitrosylation of cysteine-156 has been shown to facilitate oligomerization (Tada *et al.* 2008). The de-*S-*nitrosylation process is also receiving considerable attention, with particular focus being the roles of thioredoxins (TR). With NPR1 it is the *S-*nitrosylated cysteine-156 that is reduced by TR to promote monomer formation (Tada *et al.* 2008). These observations neatly link oxidative and salicylic acid signalling modules with NO events. In stomata, NO and oxidative events are well understood to work in tandem (Wilson *et al.* 2009) and this is clearly also the case during plant defence. Interestingly, NO is well known to influence the biosynthesis of salicylic acid (SA) so that this could promote NPR1 signalling, while NO, presumably by promoting GSNO, could also encourage oligomerization. These could be seen to be contradictory events but GSNO also promotes the NPR1–TGA1 interaction to strengthen binding to cognate promoter sequences, thereby elevating the expression of *pathogenesis-related protein* gene expression (Lindermayr *et al.* 2010).

Other subtle interactions between NO/GSNO, SA and oxidative stress are also revealed by the recent work of Yun *et al.* (2011). SA is known to drive the speed of the HR by augmenting the generation of oxidative stresses, but these authors suggested that at lower GSNO concentrations cell death was augmented by other as yet unknown mechanisms. At higher concentrations of *S*-nitrosothiol, the activity of the ROS-generating enzyme NADPH oxidase, AtRbohD, was suppressed. This was shown to arise from *S-*nitrosylation of a cysteine-890 to affect enzyme binding to its key cofactor FAD. Thus, *S-*nitrosylation of NADPH oxidase appears to be a mechanism through which the plant can regulate oxidatively driven cell death. However, other members of the NADPH gene family have other roles, for example AtRhohC in root development and root hair formation (Foreman *et al.* 2003), so this regulatory mechanism could have much wider physiological roles.

Tyrosine nitration is dependent on peroxynitrite, which is generated via superoxide and NO (O_2_^−^ + NO→ONOO^−^). This is a highly toxic molecule that can generate hydroxyl radicals (ONOO^−^ + H^+^→NO_2_^−^ + OH^.^) and cause considerable macromolecular damage via proton abstraction, and could lead ultimately to cell death as in mammalian systems (Pacher *et al.* 2007). Alternatively, NO_2_^−^ may be added at the *ortho* position on the tyrosine aromatic ring, which could exert steric effects to alter the configuration of a protein. Compared with studies on *S-*nitrosylation, there have been relatively few studies on nitration, possibly because the latter's irreversible nature made it a less attractive regulatory switch. However, one of the most elegant studies has focused on the role of nitration during the HR in *Arabidopsis*, which revealed a novel regulation of peroxiredoxins (Prx). Peroxiredoxins can detoxify the highly reactive peroxynitrite ion and this has been demonstrated by two plastid-located Prx: PrxIIE and 2-Cys-Prx (Sakamoto *et al.* 2003; Romero-Puertas *et al.* 2007). If ONOO^−^ contributes to HR-mediated cell death, then Prx would suppress this, but PrxIIE is inhibited by *S-*nitrosylation, thereby allowing the propagation of ONOO^−^ (Romero-Puertas *et al.* 2007; Cecconi *et al.* 2009). Clearly, the identification of nitrated proteins is an ongoing process, but it also highlights the need to develop an organelle-specific understanding of NO effects as, due to its reactivity, it is unlikely that ONOO^−^ will move out of the plastids to nitrate cytoplasmic proteins.

Taking the *S-*nitrosylation/nitration studies together, some tentative observations can be made. Invariably, it has been shown that NO modifies the activity of enzymes and some key signalling components, and many have suggested an integration between NO/GSNO–SA–ROS-associated events. Although this may simply reflect the research interest of the main workers in the field, it may suggest that NO effects could be exerted to a large extent by influencing the redox status of the cell. Another feature is that *S-*nitrosylation/nitration studies seem to be suggesting that NO modifies signalling pathways which have been characterized as part of the action of another signal. Thus, there are redox-associated proteins, PrxIIE (Romero-Puertas *et al.* 2007), AtRhobD (Malik *et al.* 2011), SA signalling pathway proteins salicylic acid-binding protein 3 (Wang *et al.* 2009), NPR1 (see above) (Tada 2009), TGA1 (Lindermayr *et al.* 2010) and auxin signalling TIR1 (Terrile *et al.* 2012). Other pathways such as jasmonates are also being suggested (Manjunatha *et al.* 2012; Mur *et al.* 2012). Thus, NO could also mainly act as a modifier of other signalling cascades. This was also suggested from a bioinformatic analysis of ‘NO-responsive’ promoters where salicylate- and jasmonate-responsive *cis-*elements were prominent (Palmieri *et al.* 2008). This stated, it is possible that further characterization of *S-*nitrosylative control of the R2R3-MYB class of transcription factors may identify an NO-specific transcriptional output.

Modulation of guanylate cyclase represents an NO-specific mechanism of signalling and there are many reports showing that cGMP is a facet of NO effects in plants (Klessig *et al.* 2000; Pagnussat *et al.* 2003; Szmidt-Jaworska *et al.* 2004, 2008, 2009; Suita *et al.* 2009; Li and Xue 2010; Wang *et al.* 2010; Dubovskaya *et al.* 2011). Indeed, even Viagra has been shown to have effects in plants (Siegel-Itzkovich 1999). Paradoxically, until recently, higher plants were thought to lack the required soluble form of guanylate cyclase (Schaap 2005), although it is present in single-cell algae (de Montaigu *et al.* 2010). Indeed, in *Chlamydomonas*, a soluble guanylate cyclase influenced NR expression (de Montaigu *et al.* 2010). Recently, a novel guanylate cyclase that generates cGMP and binds NO has been described (Mulaudzi *et al.* 2011). It is imperative that this be extensively characterized and integrated into our existing knowledge of NO networks.

## How does plant-generated NO fit into the nitrogen cycle economy?

An important point frequently disregarded by plant NO scientists is that plants are being continually exposed to NO from the soil. Nitric oxide production is a feature of the oxido-reductive steps ranging from NH_4_^+^ to NO_3_^−^ that form the nitrogen cycle. Various factors also influence NO production in soil such as temperature, oxygen availability, humidity, soil pH and nitrogen status. These influence the activities of nitrifying and denitrifying bacteria which under different conditions can produce NO at differing rates (Anderson and Levine 1986).

If the plant is being continuously exposed to NO, how can NO function as a discrete endogenous signalling molecule, particularly in the root? We suggest that these questions highlight the importance of *in planta* NO scavenging mechanisms as key in NO biology. Some simple chemicals such as urate have been shown to prevent NO toxicity (Alamillo and Garcia-Olmedo 2001), but more selectivity is offered by enzymatic regulation by GSNO reductase (GSNOR) (Malik *et al.* 2011) and nsHb (Gupta *et al.* 2011*b*).

Pools of reduced glutathione (GSH) are readily available within plants and GSH can be oxidized by NO to form GSNO. This represents a stable reservoir of potential NO signal so that the regulation of GSNO content represents an important regulatory step in NO regulation. The key enzyme regulating GSNO pools is GSNOR; GSNOR will reduce GSNO to ultimately produce glutathione disulfide (GSSG) and ammonia (NH_3_), and GSSG can be reduced by glutathione reductase to re-enter the GSH pool. In *Arabidopsis*, GSNOR exists as a single gene (AtGSNOR1; At5g43940), resulting in an increase in nitrosothiolates such as GSNO and thus also increases in *S-*nitrosylated proteins (Feechan *et al.* 2005). Thus, GSNOR represents an important enzyme regulating indirect NO effects via *S-*nitrosylation.

**Fig. 2:**
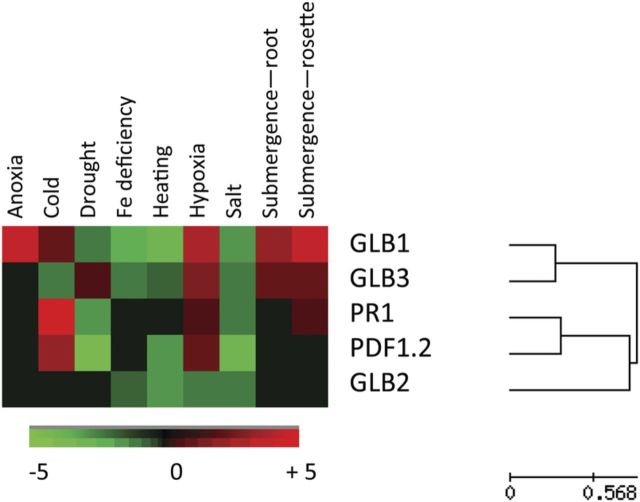
**Transcriptional regulation of *Arabidopsis* non-symbiotic Hb genes in response to abiotic stress.** Transcriptomic data for the non-symbiotic Hbs *GLB1* (At2g16060), *GLB2* (At3g10520) and *GLB3* (At4g32690) and for comparative purposes the salicylic acid marker gene *PR1* (At2g14610) and jasmonic acid marker gene *PDF1.2* (At5g44420) were extracted from the Genevestigator database (Zimmermann *et al.* 2004). The data were grouped by hierarchical cluster analysis and displayed using a heat map using Epclust software. Green represents down-regulation compared with controls and red up-regulation. Black squares indicate no net change in expression. The fold up/own regulation range is indicated.

Our interest has focused on the role of the direct oxidation of NO by nsHb and its role in regulating NO content within plants (Gupta *et al.* 2011*b*; Hebelstrup *et al.* 2012; Mur *et al.* 2012). Plant Hbs are able to regulate several of the effects of NO, as recently reviewed by Hill (2012). Plant Hbs may be sub-divided into three classes: I, II and III. Most Hbs found in association with nitrogen-fixing bacteria in root nodules of plants appear to have evolved from class II Hb, which has a relatively low affinity for O_2_ (*K*_m_ ∼ 150 nM), so that this is readily released under low partial pressures of O_2_. As such, the functions of most of those Hbs called ‘symbiotic haemoglobins’ are in facilitating oxygen supply to tissues within nitrogen-fixing nodules. However, this requires a high concentration of Hb. Other class II ‘non-symbiotic’ Hbs (nsHbs) are found in other tissues at low concentration where the contribution to facilitated oxygen diffusion is negligible (Heckmann *et al.* 2006). We have previously shown that such class II nsHbs do contribute to NO removal when over-expressed (Hebelstrup *et al.* 2006, 2012). Class III Hbs are truncated Hbs with a very low affinity for O_2_ (*K*_m_ ∼ 1500 nM) and, given their closer homology to bacterial Hbs, they may have been acquired by horizontal gene transfer (Watts *et al.* 2001). The function of these truncated Hbs is obscure and no observable phenotype was noted in *glb3*, an *Arabidopsis* class III mutant (Mur *et al.* 2012). Class I nsHbs possess very high affinity for O_2_ (2 nM) so that they represent poor oxygen carriers (Smagghe *et al.* 2009). Given this it appears that NO oxidation is an important role. During hypoxic/anoxic conditions, the oxidation of NO to NO_3_^−^ by oxyhaemoglobin [Hb(Fe^2+^)O_2_] is coupled to the reduction of NO_3_^−^ and NO_2_^−^, resulting in an Hb/NO cycle (Dordas *et al.* 2004). In this Hb/NO cycle, excess NAD(P)H is oxidized to maintain electron flow and ATP production under hypoxic conditions (Dordas *et al.* 2003; Stiomenova *et al.* 2007). Nitric oxide oxidation by Hb(Fe^2+^)O_2_ results in the formation of oxidized ferric metHb [Hb(Fe^3+^)] and so the reaction is (Hb(Fe^2+^)O_2_ + NO^+^ →Hb(Fe^3+^) + NO_3_^−^). Haemoglobin can be reduced back to its ferrous form [Hb(Fe^2+^)] by an associated reductase (2Hb(Fe^3+^) + NAD(P)H→2Hb(Fe^2+^) + NAD(P)^+^ + H^+^). The NO_3_^−^ produced is reduced to NO_2_^−^ by NR (NO_3_^−^ + NAD(P)H→NO_2_^−^ + NAD(P)^+^ + OH^−^) and subsequently by mitochondrial nitrite NO-reductase activity (Mt NINOR) at complex III and cytochrome *c* oxidase NO_2_^−^ is reduced back to NO to restart the cycle (2NO_2_^−^ + H^+^ + NAD(P)H→2NO + NAD(P)^+^ + 2OH^−^). Following this reasoning, hypoxically generated NO could improve the plant's energy status by adding to the Hb/NO cycle (Igamberdiev and Hill 2009); and possibly not only during hypoxia but also in bulky tissues where low internal oxygen is present in the centre of tissues. This, again, should be actively explored in future. Besides the Hb/NO cycle, the role of nsHb in NO removal has also attracted considerable interest. Early work used over-expressed bacterial Hb *hmpX* in transgenic lines as a useful method to reduce NO production and show the roles for NO in the HR, responses to UV-B, symbiotic interactions and senescence (Zeier *et al.* 2004; Boccara *et al.* 2005; Mishina *et al.* 2007; del Giudice *et al.* 2011; Tossi *et al.* 2011). The logical inference of this work is that nsHbs reduce endogenous NO production from plants and possibly the environment. We have provided evidence for this in our recent work where unstressed *Arabidopsis* lines with reduced expression of nsHbs [*GLB1* (At2g16060) and *GLB2* (At3g10520)] exhibited increased NO production (Hebelstrup *et al*. 2012; Mur *et al.* 2012). When NO-generating systems are deployed, it may be assumed that the NO generated by (for example) NR will swamp the scavenging capacity of nsHbs. However, some of our data have revealed an interesting regulatory mechanism whereby Hb expression (*GLB1*) is reduced in a manner which is apparently inversely correlated with the patterns of NO generation (Mur *et al.* 2012). Although we focused on responses to pathogens, interrogation of transcriptome data in the Genevestigator database (Zimmermann *et al.* 2004) has suggested that *GLB1* (the major Hb) is also suppressed in response to heat, Fe deficiency, salt and drought stress (Fig. 2). Interestingly, in stresses linked to low oxygen caused by flooding (both root and shoot expression), hypoxia or anoxia where the Hb would be expected to contribute to plant fitness via the Hb/NO cycle, *GLB1* is induced. Similarly, in response to external nitrate when both NO-generating NR and Hb would be required, the expression of genes encoding these proteins appeared to be co-regulated in maize (Trevisan *et al.* 2011). There are several indications that plant Hbs can control development and physiological reactions by modulating cellular NO levels, which interfere with the actions of various hormones (Hill 2012). For example, *Arabidopsis* plants with silencing of GLB1 (class I nsHbs) exhibit increased cellular NO levels, resulting in modified development with stunted organs, loss of apical dominance and late flowering (Hebelstrup *et al*. 2006; Hebelstrup and Jensen 2008). Thus, it would seem that coupled to characterization of the NO generation mechanism, the means through which Hb expression is regulated is as important and this is actively being investigated by ourselves and others. Comparisons of Genevestigator expression data for *GLB1* with those of the SA marker genes *PR1* and the jasmonate marker gene *PDF1.2* suggested that these defence signals were unlikely to be playing a key regulatory role (Fig. 2). Another aspect that needs considering is how far Hb could help to preserve plant nitrogen. Our recent results using *glb1* suppressed lines have suggested that in the absence of Hb, nitrogen loss during hypoxia via NO generation is substantial: ∼0.2 mmol NO_3_^−^ g FW^−1^ lost over a 24-h period (Hebelstrup *et al.* 2012).

## Conclusions and forward look

To conclude, in the course of this review we have highlighted only some basic and persistent questions. The sources of NO generation have now been extensively defined but NO generation from polyamines, HA and especially arginine has resisted elucidation. This may be because no appropriate mutants, genes or proteins have been identified. Thus, plant biologists have been lucky that NIA1 has proven to be a major source of NO despite some functional redundancy with NIA2. Thus, the *nia1* mutant exhibits reduced NO production even when NIA2 is still functional (Wilson *et al.* 2009). However, for other NO generation mechanisms, problems with lethality, functional redundancy or their activation only under precise conditions (for example, normoxia and hypoxia) may be the reason that no generation mutants have been isolated. Thus, it may be that the plant ROS field offers a salutary lesson, as here generation mechanisms have often been characterized via biochemical means. This also highlights another theme of our review, the necessity to develop a better means of measuring NO, both to assay NO generation and the site of its generation. Currently, no technique fully meets all these requirements but we have noted ongoing developments in fluorescent dyes that could ultimately provide NO scientists with a key resource.

Moving to consider NO signalling, currently a major focus is on *S-*nitrosylation and nitration events. We hope that our review of the early work suggesting that NO acts with cGMP (for example, Durner *et al.* 1998) will serve to inspire a revisiting of this possibility and may, incidentally, reveal a signalling pathway that is similar to that found in animals. Our last theme is one that is, understandably, often not considered by laboratory-based plant scientists, namely how do plant signalling pathways function in an open environment. This is particularly apposite for NO as plants are being exposed to this signal from many exogenous sources. We therefore suggest that NO scavenging, e.g. by endogenous Hb, should be considered to be as important as NO generation in understanding *in planta* NO signalling.

## Sources of funding

The work of L.A.J.M. is supported by grants from the BBRSC and the Centre for Integrated Research in the Rural Environment (CIRRE; http://www.cirre.ac.uk/), UK; that of K.H.H. by the Danish Council for Independent Research Technology and Production Sciences; that of K.J.G. by a Marie Curie Intra European Fellowship for Carrier Development within the 7th European Community Framework Programme; and that of J.M., S.M.C. and F.J.M.H. by the EU-FP6-Infrastructures-5 programme, project FP6-026183 Life Science Trace Gas Facility.

## Contributions by the authors

Most of the text of this review was written by L.A.J.M., K.H.H. and K.J.G. Comments and editorial changes on approaches to NO measurement were provided by J.M., S.M.C. and F.J.M.H., while S.P. aided L.A.J.M. in measuring *in planta* NO production following tobacco leaf infiltration with NO donors. I.E.M. and G.V.N. provided comments and edited the section on NO-associated signalling events. Overall editorial comments and changes were provided by M.A.H.

## Conflict of interest statement

None declared.
